# Does socioeconomic status modify the association between glycemic level and MRI markers of ADRD risk in middle‐aged adults?

**DOI:** 10.1002/alz.094021

**Published:** 2025-01-09

**Authors:** A. Zarina Kraal, Hugh V. McFarlane, Jeffrey D. Pyne, Indira C. Turney, Patrick J. Lao, Jessica Mazen, Adam M. Brickman, Jennifer J. Manly

**Affiliations:** ^1^ Taub Institute for Research on Alzheimer’s Disease and the Aging Brain, New York, NY USA; ^2^ James Madison University, Harrisonburg, VA USA; ^3^ Columbia University Irving Medical Center, New York, NY USA; ^4^ Department of Neurology, Columbia University, New York, NY USA

## Abstract

**Background:**

High glycemic levels, indexed by hemoglobin A1c (HbA1c), heighten risk for Alzheimer’s Disease and Related Dementias (ADRD). Previous studies suggest that high HbA1c and low socioeconomic status (SES) may be associated with MRI markers of ADRD risk, including lower cortical thickness and greater white matter hyperintensities (WMH). The weathering hypothesis suggests that the stress of low SES accelerates and exacerbates physiological deterioration, leading to worsening health outcomes. However, the role of SES as a potential moderator of the associations between HbA1c and MRI markers of ADRD is underdeveloped. We hypothesized that higher HbA1c and lower SES are associated with MRI markers of ADRD risk, and the association between HbA1c and MRI markers is stronger among individuals with low SES.

**Method:**

Participants were middle‐aged adults in the Offspring Study of Racial and Ethnic Disparities in Alzheimer’s Disease (N = 616; age = 54.5±10.6; 68.6% Latinx, 21.2% Non‐Latinx Black, 7.2% Non‐Latinx White; 65.6% women). HbA1c level was determined from blood assays. MRI markers included cortical thickness of AD signature regions and log‐transformed WMH. Confirmatory factor analysis estimated SES from self‐reported income and education. Structural equation models quantified main and interaction effects of HbA1c and SES on MRI markers, adjusting for age, sex, and prediabetes/type 2 diabetes.

**Result:**

There were no main or interaction effects of HbA1c and SES on either MRI marker, independent of covariates. However, examination of moderation by each SES indicator showed that higher HbA1c was associated with lower cortical thickness among participants with high income (unstandardized estimate = ‐0.0214, SE = 0.0107, 95%CI[‐0.0424, ‐0.0003]). Effect estimates were similar in analyses restricted to individuals with prediabetes and type 2 diabetes (n = 250; age = 58.1±9.0; 72.8% Latinx, 23.6% Non‐Latinx Black, 3.7% Non‐Latinx White; 68.8% women).

**Conclusion:**

HbA1c‐related effects on markers of ADRD risk in middle‐age may occur via neurodegenerative processes. Associations involving cerebrovascular pathways warrant further investigation. Examination of other indicators of SES (e.g., occupation, wealth, debts) and region‐specific WMH may help clarify the role of SES in modifying the link between glycemic level and brain health.

